# Sympathetic Neural Mechanisms in Hypertension: Recent Insights

**DOI:** 10.1007/s11906-023-01254-4

**Published:** 2023-07-14

**Authors:** Guido Grassi, Raffaella Dell’Oro, Fosca Quarti-Trevano, Jennifer Vanoli, Suzanne Oparil

**Affiliations:** 1 Department of Medicine and Surgery, Clinica Medica, University of Milano-Bicocca, Via Pergolesi 33, 20052 Monza, Milan, Italy; 2 Department of Medicine, School of Medicine, University of Alabama at Birmingham, Birmingham, AL 35294 USA

**Keywords:** Hypertension, Sympathetic nervous system, Heart rate, Hypertensive phenotypes, Sleep duration, Nighttime blood pressure

## Abstract

**Purpose of Review:**

To examine published and unpublished data documenting the role of sympathetic neural factors in the pathogenesis of different hypertensive phenotypes. These phenotypes relate to attended or unattended blood pressure measurements, to nighttime blood pressure profile alterations, and to resistant, pseudoresistant, and refractory hypertension. Results of original clinical studies as well as of recent meta-analyses based on the behavior of different sympathetic biomarkers in various hypertensive forms will be also discussed.

**Recent Findings:**

Studies performed in the past decade have shown that office blood pressure measurements, including in recent years those characterizing unattended or attended blood pressure assessment, are associated with profound changes in the behavior of different sympathetic biomarkers. This is the case for the clinical hypertensive phenotypes characterized by alterations in the nocturnal blood pressure profile and by sleep duration abnormalities. This is also the case for the clinical conditions defined as resistant, refractory, and pseudoresistant hypertension.

**Summary:**

Data reviewed in the present paper highlight the relevance of sympathetic neural factors in the development and progression of different clinical hypertensive phenotypes. This suggests that a common hallmark of the majority of the essential hypertensive states detectable in current clinical practice is represented by the alteration in the sympathetic blood pressure control.

## Introduction

More than a decade ago, our group in Milan published in this journal a review focused on the role of the sympathetic nervous system in the pathogenesis of essential hypertension and in the development of the related metabolic disease [1•]. During recent years, many studies have been published providing new information on the relevance of sympathetic neural mechanisms in the essential hypertensive state and in the occurrence of its complications.

The aim of the present paper is to provide an update on the new findings collected on this issue during the last decade. Following an introductory paragraph summarizing the information collected in the past on the role of the sympathetic nervous system in essential hypertension, we will address four major issues, on which a special interest has been focused due to their potential clinical implications. First, we will examine the evidence recently collected on the role of the sympathetic nervous system in determining the hemodynamic responses to office blood pressure measurements (so called “white-coat effect”) and more specifically to the attended or unattended blood pressure assessment. This will be followed by an analysis of the data collected with the aim to better define the possible relationships between sleep quality and quantity and sympathetic activation occurring in the essential hypertensive state. Third, the main features of the sympathetic profile characterizing different hypertensive phenotypes, including resistant, pseudoresistant, and uncontrolled hypertension, will be examined. We will finally describe the behavior of various sympathetic biomarkers in uncomplicated hypertension and in pathological conditions, characterized by an elevated cardiovascular risk, frequently associated with the hypertensive state, such as obesity, obstructive sleep apnea, and metabolic syndrome.

## Cornerstones of “Sympathetic” Research from 1960 to 2010

**Fig. 1:**
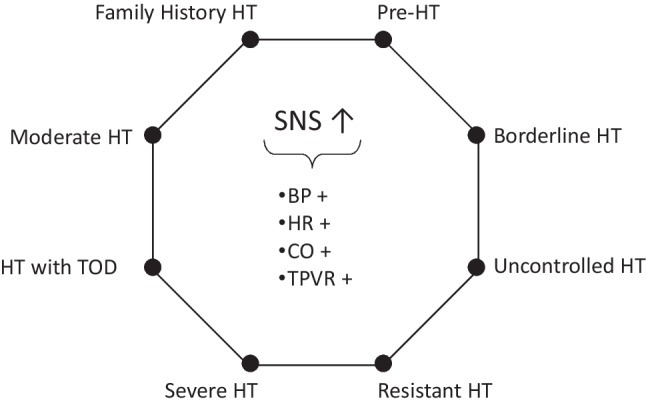
Scheme illustrating the hypertensive phenotypes characterized by sympathetic activation (SNS ↑). Cardiovascular effects of SNS activation include increase in blood pressure (BP +), heart rate (HR +), cardiac output (CO +), and total peripheral vascular resistance (TPVR +). HT hypertension, TOD target organ damage

Figure 1 summarizes the main conclusions achieved by the studies published during the past five decades up to 2010 on the participation of the sympathetic neural mechanisms at the development and progression of the essential hypertensive state [2, 3••]. Data have been collected showing that in normotensive people with a family history of hypertension, some neuroadrenergic markers are already activated. This is the case for the sympathetic nerve traffic responses, as assessed by microneurography, to an adrenergic stimuli (cold pressor test), which have been shown to be potentiated in normotensive subjects with a family history of hypertension, predisposing subjects to a blood pressure elevation in the subsequent years [4]. Similar data have been collected in young normotensive women [5] and, by assessing 24-h catecholamine urinary excretion, also in normotensive children with a family history of hypertension [6]. When the evaluation was performed in the so-called prehypertensive state, namely, the condition characterized by blood pressure values still below 140/90 mmHg but positioned in the high normal range, an already elevated sympathetic cardiovascular function was detectable via the microneurographic technique [7]. More pronounced levels of adrenergic overdrive have been shown in the hypertensive state of mild-to-moderate and more severe degree, with a significant direct relationship between the magnitude of the sympathetic activation and the amount of the blood pressure elevation [2]. Finally, when the hypertensive state is associated with target organ damage, the degree of the sympathetic overdrive appears to be potentiated, suggesting participation of neurogenic mechanisms at the occurrence of the structural and functional alterations of the macrocirculation, the microcirculation, the kidney, and the heart frequently detected particularly in the severe hypertensive state [2]. The sympathetic potentiation reported when hypertension is complicated by cardiac and renal damage is supported also by the evidence, collected via the regional norepinephrine spillover technique, that the kidneys and the heart are the organs more frequently displaying in the hypertensive condition a marked sympathetic overdrive [8•]. Finally, it should be worthy to mention that many studies have demonstrated the reversibility of the hypertension-related sympathetic overactivity via non-pharmacological and pharmacological interventions, including very recently the procedure of bilateral renal nerve ablation [9••]. Whether and to what extent the reduction in the adrenergic overdrive induced by antihypertensive treatment means a true “sympathetic normalization” or rather more simply a transfer process toward reduced (but still abnormal) sympathetic values remains to be seen. The question is of clinical relevance considering that antihypertensive drug treatment, although reducing cardiovascular risk by lowering elevated blood pressure, has been shown not to bring it back to the normotensive level [10]. This phenomenon is well known as “residual risk” of the treated hypertensive patient [10]. It is likely that many factors concur at determining this “irreversible risk,” including the abovementioned residual sympathetic activation reported in the treated hypertensive patients.

## New Achievements

As mentioned above, new information has been gained during the last decade on different pathophysiological and clinical topics, which have a common background, i.e., the involvement of the sympathetic nervous system in the blood pressure regulation and its alterations occurring in hypertension. These new data will be discussed in the following sections.

### Sympathetic Influences and Office Blood Pressure Measurement

As known since several years, office blood pressure measurement by a doctor via the sphygmomanometric technique triggers an alerting reaction in the patients by favoring a blood pressure and heart rate increase which may persist for several minutes after the visit and interfere with the evaluation of the “real” blood pressure values [11]. In order to obtain information about the participation of the sympathetic nervous system at these hemodynamic alterations, we assessed during sphygmomanometric blood pressure measurement by a doctor unfamiliar to the patients, along with beat-to-beat blood pressure and heart rate, postganglionic sympathetic nerve traffic to the muscle and skin circulation (microneurography, peroneal nerve) [12]. Evidence has been obtained that the pressor and tachycardic responses to blood pressure measurement by a doctor are mediated by complex neurogenic mechanisms, which include (1) a marked stimulation of the sympathetic nerve traffic to the skin circulation and (2) a profound sympathoinhibition at the level of the muscle vascular district [12]. Thus, skin sympathetic-mediated vasoconstriction together with vasodilatation in the skeletal muscle circulation appears to be the key features of the cardiovascular responses to doctor blood pressure measurement. This appears to mimic the so called “defense reaction” described years ago in experimental animals during exposure to an emotional stimulus such as the one occurring during fighting [13].

The information about the sympathetic responses to blood pressure measurements has been expanded in more recent years with 2 new sets of data. The first one refers to the assessment of nurse blood pressure measurements on hemodynamic as well as sympathetic variables [14]. This was done in a group of mild untreated hypertensive patients who underwent various hemodynamic and sympathetic measurements as previously described, with the difference that a nurse replaced the doctor in assessing blood pressure. Sphygmomanometric assessment of blood pressure by the nurse markedly attenuated the blood pressure and heart rate increase detected during the doctor visit [14]. This was paralleled by a significant reduction in the muscle and skin sympathetic responses to the procedure, suggesting that the attenuated “white-coat effect” seen during nurse blood pressure measurement is coupled with a reduction in the sympathetic neural responses to this emotional reaction [14].

**Fig. 2:**
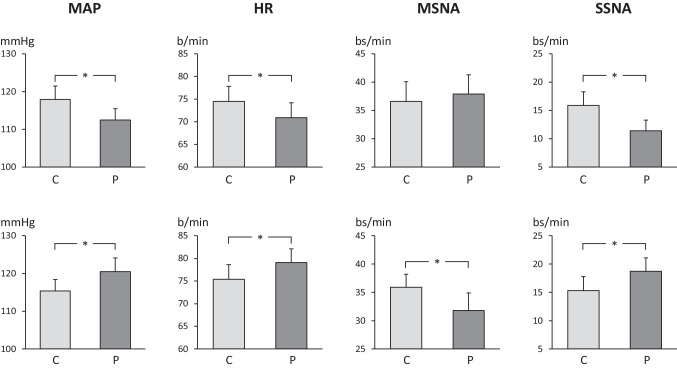
Mean arterial pressure (MAP), heart rate (HR), muscle sympathetic nerve traffic (MSNA), and skin sympathetic nerve traffic (SSNA) values before (C) and during unattended (upper panels) and attended (lower panels) blood pressure measurements. Data are shown as mean (± SEM) peak responses (P). Asterisks (*p < 0.05) refer to the statistical significance between values recorded before at during the procedure. Figure based on data of Ref 15

The second set of new data very recently collected on the sympathetic responses to office blood pressure evaluation refers to the attended or unattended blood pressure measurements [15•]. The background for this recent study is the observation that self-measurement of blood pressure by the patient without the presence of a doctor (so called “unattended blood pressure”) may provide values significantly lower than the ones collected with the same measurement procedure but in presence of the medical personnel (“attended blood pressure”) [16]. Needless to say that this blood pressure difference, which is consistent for magnitude (about 13 mmHg and 11 mmHg for systolic and diastolic, respectively), is relevant in clinical practice. Indeed, the difference may become important (1) for guiding the medical decision about threshold and target blood pressure values for the therapeutic intervention and (2) for determining which of the two blood pressures might better predict the risk of future cardiovascular events [16]. The results of the study, which have been performed in 18 untreated hypertensive patients, are illustrated in Fig. 2, which show the mean arterial pressure, heart rate, muscle, and skin sympathetic neural responses to unattended (upper panels) and attended (lower panels) automatic blood pressure measurements by the patients. In absence of the doctor, blood pressure values underwent a reduction during automatic evaluation [15•]. They were associated with a reduction in heart rate and skin sympathetic nerve traffic, with unmodified muscle sympathetic neural drive. On the other hand, in presence of the doctor, automatic measurements of blood pressure revealed an increase in the recorded values, in association with a slight heart rate and skin sympathetic nerve traffic elevation together with a slight decrease in muscle sympathetic neural outflow [15•]. Taken together, these data strongly support the notion that neuroadrenergic factors participate at, and probably are responsible for, the blood pressure changes associated with unattended and attended blood pressure measurements.

### Sympathetic Activation and Sleep Duration in Hypertension

The interest in hypertension research for the behavior of sympathetic cardiovascular function during sleep is multifold and founded on at least three major evidences. First, abnormalities in the quality/quantity of the nighttime blood pressure reduction, characterizing the non-dipping, extreme dipping and reverse dipping profile, represent strong predictors of an increased cardiovascular risk and may favor the development and progression of target organ damage [3••, 17••]. Second, sleep disordered breathing, such as obstructive sleep apnea, is frequently detected in the hypertensive patients, particularly when an overweight condition or an obese state complicates the high blood pressure state [3••]. They may favor “per se” the development during sleep of serious cardiovascular adverse events, including major life-threatening cardiac arrhythmias and sudden death [18•]. Third, in hypertensive individuals, an excessive early morning blood pressure surge during the wake-up time has been also shown to be associated with an increased risk of fatal cardiovascular events [19].

**Fig. 3:**
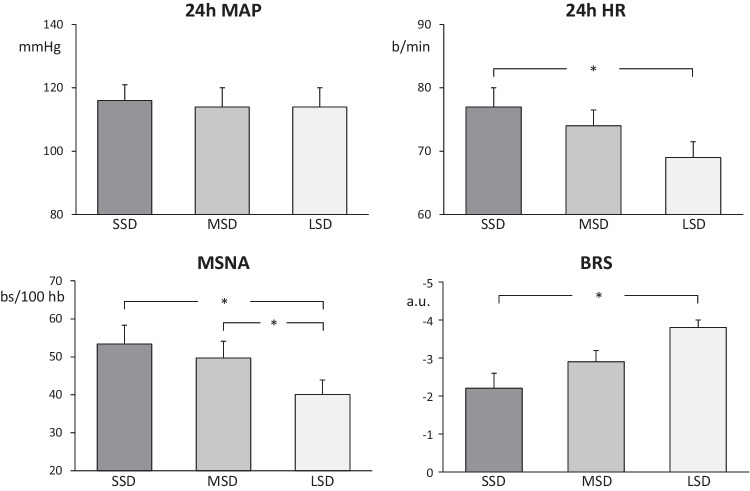
Twenty-four-hour mean arterial pressure (MAP), heart rate (HR), muscle sympathetic nerve traffic (MSNA), and baroreflex sensitivity (BRS) in patients with short sleep duration (SSD), medium sleep duration (MSD), and long sleep duration (LSD). Data are shown as mean (± SEM) values. Asterisks (*p < 0.05) refer to the statistical significance between groups

Data collected in the past years have confirmed the occurrence in almost all the above mentioned clinical phenotypes of a marked sympathetic activation, a common hallmark which supports in many of the of the abovementioned clinical conditions the close relationships between sleep disorders, hypertension, and neuroadrenergic abnormalities [2, 3••]. Recently, these relationships have been strengthened with new evidence provided by our group on the involvement of neurogenic mechanisms in the increased cardiovascular risk displayed by hypertensive patients with short sleep duration [20], a condition well known to be characterized by an increased risk of cardiovascular events [21, 22]. Microneurographic assessment of muscle sympathetic nerve traffic together with spontaneous baroreflex sympathetic sensitivity, 24-h ambulatory blood pressure, and heart rate, were performed in a group of 26 untreated moderate essential hypertensive patients [20]. They were without other cardiovascular or non-cardiovascular disease, including obstructive sleep apnea, and were subdivided in 3 groups according to sleep time duration < 6 h, between 6 and 7 h, and > than 7 h. Time sleep duration and efficiency were assessed at patients’ home via actigraphy. As shown in Fig. 3, the 3 groups, which displayed similar age (about 66 years on average), gender distribution, and body mass index (about 28 kg/m^2^), showed superimposable 24-h blood pressure values. Sympathetic nerve traffic in the peroneal nerve was significantly greater in patients with short sleep time duration than in those characterized by long sleep time, similar difference being detected in the remaining group of patients displaying a medium sleep time duration. This was the case also for 24-h heart rate values, significantly more elevated in the group with short sleep duration, suggesting that the adrenergic overdrive was detectable both at peripheral and cardiac level. Of particular interest was the observation that the sympathetic activation seen in the patients with short time sleep was accompanied by a reduced baroreflex control of sympathetic neural function, as assessed by a computerized system relating each spontaneous sympathetic neural burst to the diastolic blood pressure and the cardiac interval during which the burst was generated [20]. It thus can be concluded that short sleep duration is characterized by sympathetic and baroreflex alterations, which concur at determining the elevated cardiovascular risk profile displayed by these patients.

### Sympathetic Profile of Resistant, Pseudoresistant, and Refractory Hypertensive Phenotypes

In the past decade, the clinical relevance of resistant, pseudoresistant, and refractory hypertension has been consistently increased, also in consideration of the new therapeutic perspectives offered by bilateral renal nerve ablation [23••, 24•]. Data collected by different groups of investigators have provided unequivocal evidence that these three hypertensive phenotypes have as common link abnormalities in the sympathetic cardiovascular control [25–27]. This is particularly the case for resistant and refractory hypertension, i.e., the conditions in which 3 or more drugs or 5 or more antihypertensive agents are unable to normalize blood pressure, respectively. Indeed, by making use of the microneurographic technique, we found that muscle sympathetic nerve traffic in the peroneal nerve is more than 40% greater in hypertensive patients with resistant hypertension as compared to moderate non-resistant hypertensive state [25]. A similar level of sympathetic overdrive has been described in these patients by assessing renal sympathetic activity via the renal norepinephrine spillover technique, a finding which confirms the participation of different sympathetic cardiovascular districts at the adrenergic overdrive typical the disease [8•, 28•]. An additional finding characterizing the neural reflex profile of resistant hypertension is represented by the marked impairment of both vagal and sympathetic components detected in these patients [25, 29]. Similar results have been reported in other studies in which the assessment of the sympathetic cardiovascular drive was based either on the assay of 24-h norepinephrine urinary excretion or on the power spectral analysis of the heart rate signal [28•].

Two further sets of data collected in recent years in this research area deserve to be mentioned. The first one refers to the observation that a peculiar clinical phenotype such as the one represented by uncontrolled masked hypertension (MUCH) displays a very pronounced out-of-clinic sympathetic activation, which may favor the development and progression of this clinical condition [30]. The second new finding is related to the evidence that pseudoresistant hypertension, i.e., the clinical condition in which the presumed resistant hypertensive state depends on a variety of factors such as inadequate drug treatment, wrong drug dosage, or lack of adherence to treatment by the patient, is not associated with any direct or indirect evidence of adrenergic overdrive [27]. This latter observation underpins the selectivity of the sympathetic overactivity to peculiar clinical phenotypes of the resistant hypertensive state.

### Sympathetic Biomarkers in the High-risk Hypertensive State

Assessment of sympathetic cardiovascular influences in hypertension and in the related disease, such as obesity, metabolic syndrome, and obstructive sleep apnea, has been based on direct and indirect approaches to evaluate adrenergic function, such as microneurographic assessment of muscle sympathetic nerve traffic, assay of venous plasma norepinephrine concentration, and power spectral analysis of the heart rate signal [1•, 8•].

Because muscle sympathetic nerve traffic recording represents the more direct and sensitive approach to assess sympathetic function in humans [1•, 2], many studies and meta-analyses have evaluated in recent years the behavior of this adrenergic biomarker in hypertension and related disease. Results show that a marked increase in sympathetic neural discharge in the peroneal nerves characterizes hypertension, obesity, metabolic syndrome, and obstructive sleep apnea [31, 32•, 33•]. These findings confirm the validity of the microneurographic approach in detecting the adrenergic overdrive in all these clinical conditions even when confounding factors have been excluded.

**Fig. 4:**
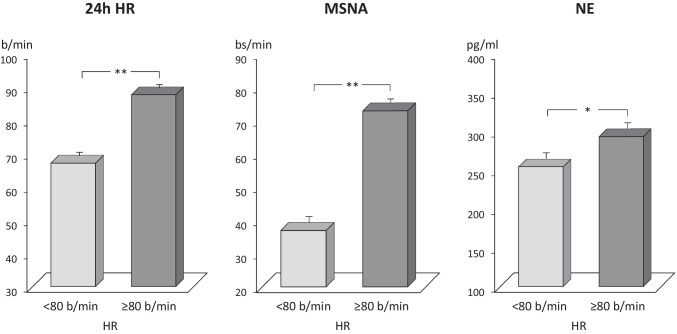
Average values (± SEM) of 24-h heart rate (24-h HR), muscle sympathetic nerve traffic (MSNA), and venous plasma norepinephrine (NE) in the groups of patients with hypertension and 24-h HR values below and above 80 bpm. Asterisks (*p < 0.002; **p < 0.0001) refer to the statistical significance between the two groups. Figure based on data of Ref 34

In the last decade, the suggestion has been advanced that information on sympathetic cardiovascular function can be obtained via a simpler method, namely, via assessment of resting heart rate values [2]. The background for this approach is multifold and includes (1) the evidence that resting heart rate values are under predominant sympathetic influences and (2) in a variety of diseases, there is significant direct relationship between the behavior of heart rate and of other markers of adrenergic drive [1•, 2]. In addition, very recent evidence has been provided that greater levels of sympathetic activation, as documented by the microneurographic technique and by the assay of venous plasma norepinephrine (Fig. 4), can be detected in hypertensive patients displaying resting heart rate > 80 beats/min [34•], i.e., the cutoff value for determining an increased cardiovascular risk indicated in the guideline document issued in 2018 by the European Society of Cardiology/European Society of Hypertension [17••]. This finding underlines once more the relevant participation of the adrenergic activation at determining cardiovascular risk profile in the hypertensive patients. It is of interest to note that similar cutoff values of heart rate values as expression of elevated adrenergic drive have been detected in obesity, metabolic syndrome, and heart failure, i.e., all clinical conditions characterized as common hallmark by a state of adrenergic overactivity.

## Conclusions

The large amount of new data collected during the past decade on the behavior of sympathetic mechanisms in the pathophysiology of different hypertensive phenotypes, including those characterizing clinical forms of resistant hypertension and those detected during the nighttime period, have permitted to gain new insights on the role of neuroadrenergic factors in the development of the hypertensive state. Both pharmacological and non-pharmacological therapeutic interventions (including recently bilateral renal nerves ablation) should thus address this neurogenic abnormality, thereby allowing not only to decrease elevated blood pressure values but also by counteracting the sympathetic overdrive to reduce an elevated cardiovascular risk.

## References

[1] Grassi G, Seravalle G, Dell’Oro R, Mancia G (2011). Sympathetic mechanisms, organ damage and antihypertensive treatment. Curr Hypertens Rep.

[2] Grassi G (2009). Assessment of sympathetic cardiovascular drive in human hypertension: achievements and perspectives. Hypertension.

[3] Oparil S, Acelajado MC, Bakris G, Berlowitz D, Cifkowa R, Dominiczak A (2018). Hypertension Nature Reviews/Disease Primers.

[4] Noll G, Wenzel RR, Schneider M, Oesch V, Binggeli C, Shaw S (1996). Increased activation of sympathetic nervous system and endothelin by mental stress in normotensive offspring of hypertensive parents. Circulation.

[5] Greaney JL, Mathhews EL, Wenner MM (2015). Sympathetic reactivity in young women with a family history of hypertension. Am J Physiol.

[6] Ferrara LA, Moscato TS, Pisanti N, Marotta T, Krogh V, Capone D (1988). Is the sympathetic nervous system altered in children with familial history of arterial hypertension?. Cardiology.

[7] Seravalle G, Lonati L, Buzzi S, Cairo M, Quarti Trevano F, Dell'Oro R (2015). Sympathetic nerve traffic and baroreflex function in optimal, normal, and high-normal blood pressure states. J Hypertens.

[8] Esler MD (2014). Sympathetic nervous system moves toward center stage in cardiovascular medicine. Hypertension.

[9] Biffi A, Dell’Oro R, Quarti-Trevano F, Cuspidi C, Corrao G, Mancia G (2023). Effects of renal denervation on sympathetic nerve traffic and correlates in drug-resistant and uncontrolled hypertension: a systematic review and meta-analysis. Hypertension.

[10] Zanchetti A (2009). Bottom blood pressure at bottom cardiovascular risk? How far can cardiovascular risk be reduced?. J Hypertens.

[11] Mancia G, Bertinieri G, Grassi G, Parati G, Pomidossi G, Ferrari A (1983). Effects of blood pressure measurements by the doctor on patient’s blood pressure and heart rate. Lancet.

[12] Grassi G, Turri C, Vailati S, Dell’Oro R, Mancia G (1999). Muscle and skin sympathetic nerve traffic during the “white-coat” effect. Circulation.

[13] Folkow B. Physiology of behaviour and blood pressure regulation in animals. In: Julius S, Bassett DR, eds. Handbook of Hypertension, vol. 9: Behavioral Factors in Hypertension. Amsterdam:Elsevier Science Publishers; 1987:pp.1–18.

[14] Grassi G, Seravalle G, Buzzi S, Magni L, Brambilla G, Quarti-Trevano F (2013). Muscle and skin sympathetic nerve traffic during physician and nurse blood pressure measurement. J Hypertens.

[15] Grassi G, Quarti-Trevano F, Seravalle G, Dell’Oro R, Vanoli J, Perseghin G (2021). Sympathetic neural mechanisms underlying attended and unattended blood pressure measurement. Hypertension.

[16] Bauer F, Seibert FS, Rohn B, Bauer KAR, Rolshoven E, Babel N, Westhoff TH (2018). Attended versus unattended blood pressure measurement in a real life setting. Hypertension.

[17] Williams B, Mancia G, Spiering W, Agabiti-Rosei E, Azizi M, Burnier M (2018). 2018 ESC/ESH guidelines for the management of arterial hypertension. Eur Heart J.

[18] Makarem N, Alcantara C, Williams N, Bello NA, Abdalla M (2021). Effect of sleep disturbances on blood pressure. Hypertension.

[19] Kario K (2010). Morning surge in blood pressure and cardiovascular events. Hypertension.

[20] Seravalle Gl, Quarti-Trevano F, Airoldi F, Bertoli S, Mancia G, Grassi G. Behavior of indirect and direct indices of sympathetic activity in relation to sleep time duration in untreated mild-to-moderate essential hypertensives . J Hypertens. 2019;37(e suppl): e39 (abstract).

[21] Cappuccio FP, Cooper D, D'Elia L, Strazzullo P, Miller MA (2011). Sleep duration predicts cardiovascular outcomes: a systematic review and meta-analysis of prospective studies. Eur Heart J.

[22] Dong J, Zhang Y, Qin L (2013). Obstructive sleep apnea and cardiovascular risk: meta-analysis of prospective cohort studies. Atherosclerosis.

[23] Acelajado MC, Hughes ZH, Oparil S, Calhoun DA (2019). Treatment of resistant and refractory hypertension. Circ Res.

[24] Matanes F, Khan MB, Siddiqui M, Dudenbostel T, Calhoun D, Oparil S (2022). An update on refractory hypertension. Curr Hypertens Rep.

[25] Grassi G, Seravalle G, Brambilla G, Pini C, Alimento M, Facchetti R (2014). Marked sympathetic activation and baroreflex dysfunction in true resistant hypertension. Int J Cardiol.

[26] Dudenbostel T, Acelajado MC, Pisoni R, Li P, Oparil S, Calhoun D (2015). Refractory hypertension: evidence of heightened sympathetic activity as a cause of antihypertensive treatment failure. Hypertension.

[27] Dell’Oro R, Quarti-Trevano F, Seravalle G, Zanchettin F, Bertoli S, Airoldi F (2019). Sympathetic nerve traffic and arterial baroreflex function in apparent drug-resistant hypertension. Hypertension.

[28] Kiuchi MG, Esler MD, Fink GD, Osborn JW, Banek CT, Böhm M (2019). Renal denervation update from the International Sympathetic Nervous System Summit:JACC state-of-the art review. J Am Coll Cardiol.

[29] Freitas IMG, de Almeida LB, Pereira NP, Mira PAC, de Paula RB, Martinez DG, et al. Baroreflex gain and vasomotor sympathetic modulation in resistant hypertension. Clin Auton Res. 2017;27:175–184. 10.1007/s10286-017-0417-7 28386627

[30] Siddiqui M, Judd EK, Jaeger BC, Bhatt H, Dudenbostel T, Zhang B (2019). Out-of-clinic sympathetic activity is increased in patients with masked uncontrolled hypertension. Hypertension.

[31] Grassi G, Pisano A, Bolignano D, Seravalle G, D’Arrigo G, Quarti-Trevano F (2018). Sympathetic nerve traffic activation in essential hypertension and its correlates. Systematic reviews and meta-analyses Hypertension.

[32] Grassi G, Biffi A, Quarti Trevano F, Dell’Oro R, Corrao G, Mancia G (2019). Sympathetic neural overdrive in the obese and overweight state: meta-analysis of published studies. Hypertension.

[33] • Biffi A, Quarti-Trevano F, Bonzani M, Seravalle G, Corrao G, Mancia G, et al. Neuroadrenergic activation in obstructive sleep apnoea syndrome. A new selected meta-analysis revisited. J Hypertens*.* 2022:40:15–23. **Meta-analysis of the studies on SNS insleep apnea.** 10.1097/HJH.0000000000003045 PMC10871617 34857700

[34] Grassi G, Quarti-Trevano F, Seravalle G, Dell’Oro R, Facchetti R, Mancia G (2020). Association between the European Society of Cardiology/European Society of Hypertension heart rate thresholds for cardiovascular risk and neuroadrenergic markers. Hypertension.

